# Severity of COVID-19 in children with cancer: Report from the United Kingdom Paediatric Coronavirus Cancer Monitoring Project

**DOI:** 10.1038/s41416-020-01181-0

**Published:** 2020-12-10

**Authors:** Gerard C. Millen, Roland Arnold, Jean-Baptiste Cazier, Helen Curley, Richard G. Feltbower, Ashley Gamble, Adam W. Glaser, Richard G. Grundy, Lennard Y. W. Lee, Martin G. McCabe, Robert S. Phillips, Charles A. Stiller, Csilla Várnai, Pamela R. Kearns

**Affiliations:** 1 Cancer Research UK Clinical Trials Unit, Institute of Cancer and Genomic Sciences, College of Medical and Dental Sciences, University of Birmingham, Birmingham, B15 2TT UK; 2 Department of Paediatric Oncology, Birmingham Children’s Hospital, Steelhouse Lane, Birmingham, B4 6NH UK; 3 Institute of Cancer and Genomic Sciences, College of Medical and Dental Sciences, University of Birmingham, Birmingham, B15 2TT UK; 4 Centre for Computational Biology, University of Birmingham, Edgbaston, Birmingham, UK; 5 Leeds Institute for Data Analytics (LIDA), School of Medicine, University of Leeds, Leeds, LS2 9JT UK; 6 Children’s Cancer and Leukaemia Group (CCLG), Leicester, LE1 7GB UK; 7 Professor of Paediatric Oncology and Late Effects Medicine, Leeds Institute of Medical Research, University of Leeds, Leeds, LS2 9JT UK; 8 Children’s Brain Tumour Research Centre, School of Medicine, The University of Nottingham, Nottingham, NG7 2UH UK; 9 Institute of Cancer and Genomic Sciences, University of Birmingham, Edgbaston, Birmingham, UK; 10 Division of Cancer Sciences, University of Manchester, Manchester Academic Health Science Centre, Manchester, M13 9PL UK; 11 National Cancer Registration and Analysis Service, Public Health England, London, SE1 8UG UK; 12 Centre for Reviews and Dissemination, University of York, York, UK; 13 Department of Paediatric Oncology, Leeds Children’s Hospital, Leeds, UK; 14 Centre for Computational Biology, University of Birmingham, Birmingham, B15 2TT UK; 15 Cancer Research UK Clinical Trials Unit, NIHR Birmingham Biomedical Research Centre, Institute of Cancer and Genomic Sciences, College of Medical and Dental Sciences, University of Birmingham, Birmingham, B15 2TT UK

**Keywords:** SARS-CoV-2, Paediatric cancer

## Abstract

**Background:**

Children with cancer are frequently immunocompromised. While children are generally thought to be at less risk of severe SARS-CoV-2 infection than adults, comprehensive population-based evidence for the risk in children with cancer is unavailable. We aimed to produce evidence of the incidence and outcomes from SARS-CoV-2 in children with cancer attending all hospitals treating this population across the UK.

**Methods:**

Retrospective and prospective observational study of all children in the UK under 16 diagnosed with cancer through data collection from all hospitals providing cancer care to this population. Eligible patients tested positive for SARS-CoV-2 on reverse transcription polymerase chain reaction (RT-PCR). The primary end-point was death, discharge or end of active care for COVID-19 for those remaining in hospital.

**Results:**

Between 12 March 2020 and 31 July 2020, 54 cases were identified: 15 (28%) were asymptomatic, 34 (63%) had mild infections and 5 (10%) moderate, severe or critical infections. No patients died and only three patients required intensive care support due to COVID-19. Estimated incidence of hospital identified SARS-CoV-2 infection in children with cancer under 16 was 3%.

**Conclusions:**

Children with cancer with SARS-CoV-2 infection do not appear at increased risk of severe infection compared to the general paediatric population. This is reassuring and supports the continued delivery of standard treatment.

## Background

Intensive treatment of children with cancer delivered by robust networks of national and international clinical trial consortia has led to marked improvements in survival over the last four decades.^1^ Therapy frequently results in significantly compromised immunity with infection being a major cause of mortality.^2–6^ Data from China, corroborated by other countries, suggest that adults with cancer are more at risk of contracting SARS-CoV-2 than the general population and are more likely to suffer from a severe form of the COVID-19 illness.^7–10^ Other datasets have shown that in a non-immunocompromised population, children are less severely affected if they contract SARS-CoV-2 than adults.^11,12^

While the impact of SARS-CoV-2 in this population is yet to be defined, the international paediatric oncology community has acted at pace to provide guidance and support to childhood cancer patients to minimise their risk of infection during the pandemic.^13,14^ However, the emerging evidence base for this is understandably limited with data restricted to individual case reports or series from single institutions as well as some incomplete regional or national datasets.^15–18^

To characterise the effects of COVID-19 in children with cancer who have attended hospital on a population-wide basis, the network of specialist hospitals managing children with cancer across the United Kingdom (UK) and the Children’s Cancer and Leukaemia Group (CCLG) established the UK Paediatric Coronavirus Cancer Monitoring Project (https://ukcoronaviruscancermonitoring.com/paediatrics/), based on the same platform as the UK Coronavirus Cancer Monitoring Project that recently reported data on adult cancer.^19,20^ We present the initial data from this project up to the end of July 2020, spanning the initial peak of the COVID-19 pandemic in the UK

## Methods

All children and adolescents under the age of 16 years with cancer in the UK are managed by one of 20 Principal Treatment Centres (PTCs). All PTCs are represented within an umbrella organisation—the Children’s Cancer and Leukaemia Group (CCLG). All twenty PTCs in the UK were invited to participate in the study (the single CCLG-affiliated centre outside the UK was excluded). The project was launched on 7 April 2020 with the facility for both prospective and retrospective data collection. Data were collected up to and including the 31 July 2020.

All SARS-CoV-2-positive cases were identified by confirming the presence of viral RNA in respiratory swabs by reverse transcription polymerase chain reaction (RT-PCR), according to the appropriate national public health guidance. RT-PCR was not mandated by the protocol.

The UK Paediatric Coronavirus Cancer Monitoring Project database was designed as a public health surveillance registry to support rapid clinical decision making, in accordance with the UK Policy Framework for Health and Social Care Research, the UK National Research Ethics Service, and the UK Governance Arrangement for Research Ethic Committees. At an institutional level, this cohort study was approved according to local information governance processes.

Each participating centre had a designated local Emergency Response Reporting Individual (ERRI) who was responsible for data collection at a local level and was supported by a Local Emergency Response Reporting Group (LERRG). All information was de-identified at source to ensure strict anonymity when shared with the research team. Data were submitted via the research electronic data capture (REDCap) database that is browser based and metadata derived.^21^ REDCap is hosted by the Institute of Translational Medicine at the University of Birmingham, Birmingham, UK.^22^

Data were collected on age, sex, ethnicity, presentation with COVID-19, severity of COVID-19 (Table 1), type of malignancy and treatment to date as well as clinical outcomes including survival and need for ongoing hospital-based care.

**Table 1:** Coronavirus severity index.

Asymptomatic	Asymptomatic but positive diagnostic test (undertaken for other reasons).
Mild	Symptoms of acute upper respiratory tract infection, including fever, fatigue, myalgia, cough, sore throat, runny nose and sneezing. Clear chest on auscultation. Some cases may have no fever, or have only digestive symptoms such as nausea, vomiting, abdominal pain and diarrhoea.
Moderate	Signs of pneumonia including fever, cough and crepitations. No increased work of breathing. Some cases may have no clinical signs and symptoms, but chest CT shows lung lesions, which are subclinical.
Severe	Respiratory distress—respiratory rate ≥70 breaths per min for infants aged <1 year, ≥50 breaths per min for children >1 year. Oxygen saturation less than 92%. Dehydration requiring intravenous fluid support.
Critical	Acute respiratory distress syndrome (ARDS) or respiratory failure requiring ventilator support. Signs of shock, encephalopathy, myocardial injury or heart failure, coagulation dysfunction or acute kidney injury. Need for ICU support for other reasons.

Adapted from Dong et al. and Qiu et al.^11,12^

Initial contact was made with the ERRI at each centre by the Steering Committee to encourage reporting of cases and completeness of datasets. This was followed with two subsequent email communications. Some care of childhood cancer patients in the UK is provided by general paediatricians in Paediatric Oncology Shared Care Units (POSCUs) under the oversight of a PTC. POSCU clinicians were given access to submit data, which was linked to their PTC, so that patients testing positive for SARS-CoV-2 at a POSCU were captured while ensuring that data were not duplicated.

Paediatric patients who had received an allogeneic stem cell transplant were excluded as this data were being separately collected by the European Society for Blood and Marrow Transplantation as part of their study looking at SARS-CoV-2 in post-transplant patients (https://www.ebmt.org/covid-19-and-bmt).

### Role of the funding source

The funder of the study had no role in study design, data collection, data analysis, data interpretation or writing of the report. GM had full access to all data from the study and had the final responsibility for the decision to submit for publication.

## Results

Up to 31 July 2020, 54 children under the age of 16 years at the time of testing were identified to have SARS-CoV-2 by PCR-based testing and had complete datasets submitted. At the time of reporting, the median observation time from positive SARS-CoV-2 test was 116 days (range 1–156 days). 98% of all patients have a minimum of 4 weeks of follow-up data from their initial positive test result.

Demographics of the patients are shown in Table 2. There was a near equal sex ratio (29 boys:25 girls). The median age at positive SARS-CoV-2 test was 5 years 0 months (range 10 months to 15 years, 9 months).

**Table 2:** Patient demographics and underlying diagnosis.

Characteristic	Number	%
Sex		
Male	29	53.7
Female	25	46.3
Ethnicity		
White	28	51.2
Black	4	7.4
Asian	8	14.8
Other	6	11.1
Unavailable	8	14.8
Diagnosis		
Acute lymphoblastic leukaemia	24	44.4
Acute myeloid leukaemia	4	7.4
CNS tumour	5	9.3
Neuroblastoma	6	11.1
Sarcomas	4	7.4
Wilms tumour	2	3.7
Hepatoblastoma	2	3.7
Retinoblastoma	2	3.7
Hodgkin lymphoma	1	1.9
Burkitt lymphoma	1	1.9
Others/non-malignant	3	5.6
Total patients	54	100

Sarcoma subtypes—1 × rhabdomyosarcoma, 2 × osteosarcoma, 1 × soft tissue sarcoma. Other tumours—1 × kaposiform haemangioendothelioma, 1 × haemophagocytic lymphohistiocytosis, 1 × paraganglioma.

The most common cancer diagnostic group was acute leukaemia; 44% of all patients had a diagnosis of acute lymphoblastic leukaemia (ALL). 52% of all girls in the study had ALL versus 38% of boys.

Thirty-three percent of children/young people with cancer identified as having SARS-CoV-2 in the UK were from a black or minority ethnic background (BAME).

### Presentation with COVID-19

**Fig. 1:**
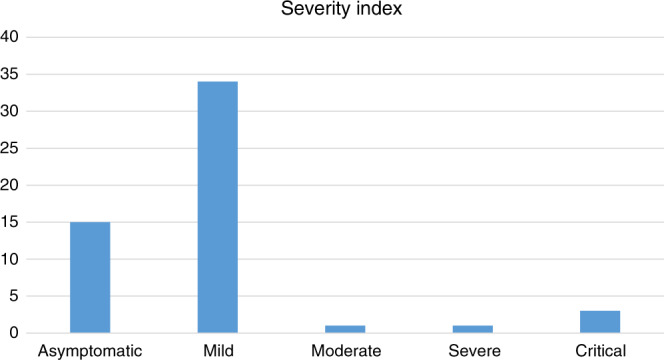
Severity index of patients presenting with SARS-CoV-2 infection. Each bar shows the absolute number of patients with SARS-CoV-2 infection at each level of severity defined in Table 1.

The overwhelming majority of patients had mild or asymptomatic infections with SARS-CoV-2 (Fig. 1). Eighty-seven percent of all symptomatic patients presented with fever while 62% had either cough or coryzal symptoms at presentation. Ten percent of patients had GI symptoms at presentation.

Only five patients had more than asymptomatic/mild infection (one moderate, one severe and three critical). All of these patients were aged under 10 and had diagnoses of ALL (*n* = 2, including one relapse), hepatoblastoma (one), osteosarcoma (one) and neuroblastoma (one). Three patients required intensive care support including one who had a background of chronic lung disease. All five patients recovered from their SARS-CoV-2 infection and were either discharged from hospital or remain in secondary care for reasons unrelated to the infection.

### Admission to hospital

**Fig. 2:**
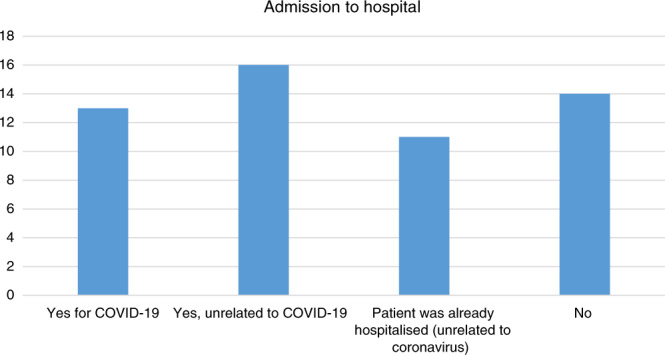
Requirement for admission to hospital for patients with SARS-CoV-2 infection. Each bar represents the need for admission to hospital and the relationship between this and SARS-CoV-2 decided by the local team.

The majority of patients were not admitted to hospital specifically for their SARS-CoV-2 infection based on the clinical review of the local team (see Fig. 2). Of those who were admitted for non-SARS-CoV-2 reasons, the majority had a fever and were initially admitted for intravenous antibiotics while awaiting the outcome of a febrile neutropenia screen. It is also common practice in some PTCs in the UK for children who present with non-neutropenic fever and are clinically well to be treated as outpatients with oral antibiotics, reducing the need for hospital admission.

### Treatment received

Treatment data were available for 38/54 patients (70%). Of those with available data, 21% were receiving very myelosuppressive chemotherapy (either induction/delayed intensification chemotherapy for ALL or chemotherapy for AML). Twenty-nine percent of patients were receiving less intensive chemotherapy (either maintenance chemotherapy for ALL/RMS or weekly vincristine). The remaining 50% of patients were receiving a range of other more standard chemotherapy regimens. Twenty-six percent of patients were receiving targeted or immune therapy with or without conventional chemotherapy including dinutuximab beta, inotuzumab ozogamicin, blinatumomab, gemtuzomab ozogamicin and larotrectinib. No patients had received high dose chemotherapy or autologous stem cell support within the 28 days preceding their positive SARS-CoV-2 result.

Of those patients with moderate, severe or critical infections, one was receiving relapse treatment, one was having delayed intensification, two were having other chemotherapy and one was having targeted therapy with blinatumomab.

### Outcome data

The majority of patients (46 (85%)) did not require admission to hospital or were admitted for a period of time and subsequently discharged following their diagnosis with COVID-19. Seven (13%) patients remained in hospital for reasons unrelated to their COVID-19. Only one patient (2%) died who was on a palliative pathway prior to the onset of COVID-19. Cause of death was progressive disease rather than from COVID-19.

## Discussion

To the best of our knowledge, the UK Paediatric Coronavirus Cancer Monitoring Project is the first national hospital whole population-based registry of paediatric patients with cancer with confirmed SARS-CoV-2 to be reported internationally.

Our data have a similar spectrum of severity to those published from a case series of children from across China with COVID-19 reported to the Chinese Centre for Disease Control and Prevention in January and February 2020 and is in keeping with the reporting emerging from other countries. The Chinese report showed that 5.6% patients had either severe or critical illness, which compared to 6.6% in our data and 8% in a recent pan-European study of COVID-19 in children. The results suggest that children and young people with cancer who contract COVID-19 are not at any more risk of serious infection than children in the general population.^12,15–18,23^ However, it is worth noting the low incidence in this population overall which may be related to the strict implementation of stringent shielding advice at the beginning of the outbreak due to the uncertainty about the severity of the infection in immunocompromised children.

Data released by Public Health England show that approximately 1460 children with cancer aged under 16 years at diagnosis are treated with systemic anticancer therapy each week in England.^24^ Extrapolating these data, we estimate that UK-wide there are currently ~1700 children under the age of 16 years in the UK undergoing chemotherapy treatment for cancer on any week in time. Since the first report of SARS-CoV-2 in the UK, we have collected data on 54 childhood cancer patients. As all PTCs across the UK participated in the study and we verified that all patients attending hospital who were SARS-CoV-2 positive were included, therefore our cohort approximates to 3% of the population of childhood cancer patients receiving systemic anticancer therapy per week. This is similar to that reported from a large hospital in Madrid.^17^ ALL accounted for 44% of children in this study, compared with around one quarter of all childhood cancer incidence, but the duration of treatment for ALL is considerably longer than for most other childhood malignancies and therefore we would anticipate proportionately there would be more children with ALL on treatment in a fixed time-period.

The median time between positive test for SARS-CoV-2 and the data cut-off date of 31 July is 116 days. We believe that it is therefore unlikely that the outcome from infection for any patient within our cohort will now change.

We had a similar ratio of girls to boys reported in our study with a small male excess. This is similar to the series reported by the Chinese Centre for Disease Control and Prevention, in which there was no significant difference in rate of COVID-19 between boys and girls.^12^ Our data are in stark contrast to the report from Memorial Sloan Kettering demonstrating 85% of all patients with a positive result were boys.^25^ It is difficult to explain the marked discrepancy; however, our results are much closer to the UK incidence figures for children with cancer, which show a small male excess.^26^

The incidence of SARS-CoV-2 infection in children from black and minority ethnic populations in our study is in keeping with the general literature, which suggests that there may be an increased infection risk for these individuals. The most recent published ethnicity data for children with cancer in the UK had an incidence of 9% in children from BAME backgrounds.^27^ The numbers of SARS-CoV-2 cases we have reported are small and the ethnicity data are incomplete. In addition, the most recent published incidence data for ethnicity in children with cancer is more than 12 years old and the demographics of the wider population may have changed in that time. However, our ethnicity finding is in keeping with that reported in other populations of a higher incidence of SARS-CoV-2 infection in non-white individuals.^28,29^

Figure 2 details the requirement for admission to hospital, which was decided by the treating oncology team. The local oncology team did not ascribe COVID-19 as the reason for admission in 16 out of 54 patients. In four cases, the admission was routine and the COVID-19 swab was identified as part of an asymptomatic screening process. However, 75% (12 out of 16) had an isolated fever on admission. These patients with an isolated fever were admitted to be monitored for signs of serious bacterial infections and, when deemed clinically fit for discharge, were allowed to go home, in accordance with routine practice for children with a fever on chemotherapy. While the admitting team did not ascribe the fever to COVID-19, it is clearly a recognised symptom. It is possible that some patients were incorrectly coded as requiring admission to hospital, which was unrelated to SARS-CoV-2; however, none of this cohort developed severe symptoms or required ICU support. Even if the fever in these patients was COVID-19 related, this is still consistent with the conclusion that children with cancer who contract COVID-19 are not at more risk of severe infection than the general population.

The patients in our dataset had a range of chemotherapy treatments in the 4 weeks preceding their SARS-CoV-2 infection including more intensive chemotherapy regimens such as induction or delayed intensification regimens for ALL. Although it is difficult to accurately define intensity of chemotherapy, we observed no trend towards more serious infection in those who received the more intensive types of therapy, although the numbers are small so it is difficult to draw definitive conclusions.

The most important strength of our study is that the design, alongside the organisational structure of healthcare delivery for children with cancer across the UK and the 100% participation of PTCs means there is a high level of certainty of comprehensive case ascertainment of SARS-CoV-2 infections identified by hospitals across the UK in those aged under 16 years receiving systemic anticancer therapy for malignancy. There will be an underestimation of the proportion of patients under 16 years of age with cancer who have contracted SARS-CoV-2 who have been asymptomatic or, at most, suffered mild symptoms. However, carers of children with cancer are well educated in the need to contact their PTC or POSCU if their child develops any symptoms. Consequently, we would expect all patients on active treatment who were more severely affected to have been reviewed in hospital and tested for SARS-CoV-2.

One limitation of our study is that, for the duration of the study, there has not been a uniform testing policy across the UK. At the beginning of the data collection period, only patients with symptoms consistent with SARS-CoV-2 who required admission to hospital were being routinely tested. Over the course of the study, this has changed so that all patients requiring admission to hospital for any reason are tested as well as those who require elective surgical procedures. These changes will have happened at different time points in different PTCs around the UK and at different points in the devolved nations. Although this study was carried out across all UK paediatric oncology centres, the number of positive patients is still small. The study is therefore prone to potential selection bias, exacerbated by small numbers and the changing policy on testing individuals throughout the study period.

Another possible limitation relates to the test for diagnosis of SARS-CoV-2 infection. In our study, all patients were diagnosed with a positive RT-PCR result from a combined nose/throat swab. There have been well documented concerns about the accuracy of this form of test, particularly regarding the sensitivity.^30^ In addition, the indication for performing a test which was based on national public health advice has changed frequently through the course of the study. These factors likely combine to mean that there is a greater proportion of children with cancer in the population who have been infected with SARS-CoV-2 but have either not been unwell enough to present to hospital or have had a negative PCR.

While this paper focuses on the risks to children and adolescents with cancer who develop proven SARS-CoV-2 infection rather than the risk of being infected, the results have informed precautionary advice given to healthcare professionals and parents. The initial advice was highly protective of those thought to be at high risk due to their immunocompromised status. The data presented here are reassuring that children with cancer are at no greater risk of severe disease than the general paediatric population and is in keeping with emerging International data. Consequently, national bodies have been able to ease some of the more stringent recommendations for patients in this population.^31^

## Conclusion

We present data from the first published national population registry of paediatric oncology patients attending hospital with SARS-CoV-2 infection. Although numbers are small, the data suggest that this group of patients are not at high risk of severe infections, similar to what has been observed in children in the wider population. These population-based data confirm the initial reports released early in the pandemic. It provides further evidence of the importance of continuing to provide routine cancer care to children and young people with malignancies in the time of COVID-19 and provides a model for how future registries could be developed for rapidly evolving conditions in populations affected by rare diseases.

## Data Availability

All data in the study can be made available at the request of the editors. At the conclusion of the study, we plan to make the data available in a publicly available database.
